# A Cross-Species Analysis of a Mouse Model of Breast Cancer-Specific Osteolysis and Human Bone Metastases Using Gene Expression Profiling

**DOI:** 10.1186/1471-2407-11-304

**Published:** 2011-07-20

**Authors:** Anguraj Sadanandam, Mitsuru Futakuchi, Costas A Lyssiotis, William J Gibb, Rakesh K Singh

**Affiliations:** 1 Department of Pathology and Microbiology, University of Nebraska Medical Center, Omaha, NE 68198-5900, USA; 2 Department of Molecular Toxicology, Graduate School of Medical Sciences, Nagoya City University, Nagoya 467-8601, Japan; 3 Department of Systems Biology, Harvard Medical School, Boston, MA 02115, USA; 4 Division of Signal Transduction, Beth Israel Deaconess Medical Center, Boston, MA 02115, USA; 5 Department of Life Sciences, Lawrence Berkeley National Lab, Berkeley, CA 94702, USA; 6 Swiss Institute for Experimental Cancer Research (ISREC), Ecole Polytechnique Federale de Lausanne (EPFL), CH-1015 Lausanne, Switzerland

**Keywords:** Osteolysis, bone metastasis, tumor-bone microenvironment, thiazide

## Abstract

**Background:**

Breast cancer is the second leading cause of cancer-related death in women in the United States. During the advanced stages of disease, many breast cancer patients suffer from bone metastasis. These metastases are predominantly osteolytic and develop when tumor cells interact with bone. *In vivo *models that mimic the breast cancer-specific osteolytic bone microenvironment are limited. Previously, we developed a mouse model of tumor-bone interaction in which three mouse breast cancer cell lines were implanted onto the calvaria. Analysis of tumors from this model revealed that they exhibited strong bone resorption, induction of osteoclasts and intracranial penetration at the tumor bone (TB)-interface.

**Methods:**

In this study, we identified and used a TB microenvironment-specific gene expression signature from this model to extend our understanding of the metastatic bone microenvironment in human disease and to predict potential therapeutic targets.

**Results:**

We identified a TB signature consisting of 934 genes that were commonly (among our 3 cell lines) and specifically (as compared to tumor-alone area within the bone microenvironment) up- and down-regulated >2-fold at the TB interface in our mouse osteolytic model. By comparing the TB signature with gene expression profiles from human breast metastases and an *in vitro *osteoclast model, we demonstrate that our model mimics both the human breast cancer bone microenvironment and osteoclastogenesis. Furthermore, we observed enrichment in various signaling pathways specific to the TB interface; that is, TGF-β and myeloid self-renewal pathways were activated and the Wnt pathway was inactivated. Lastly, we used the TB-signature to predict cyclopenthiazide as a potential inhibitor of the TB interface.

**Conclusion:**

Our mouse breast cancer model morphologically and genetically resembles the osteoclastic bone microenvironment observed in human disease. Characterization of the gene expression signature specific to the TB interface in our model revealed signaling mechanisms operative in human breast cancer metastases and predicted a therapeutic inhibitor of cancer-mediated osteolysis.

## Background

Bone is one of the most common sites for metastasis in human breast cancer. Bone metastasis results in cancer-related pain, pathological fracture, hypercalcemia, neurological defects, and immobility; all of which increase the risk of mortality and decrease the quality of life for breast cancer patients [1-4]. While a number of strategies exist to treat breast cancer bone metastases (e.g., surgery, radiation and/or chemotherapy), none are curative. Furthermore, these treatment methods have limited efficacy due in part to the fact that they do not effectively target the interaction between tumor cells and bone [5]. Even though the bisphosphonate class of drugs (which target the tumor-bone interface) have been shown to improve the quality of life and disease-free survival in some patients, more therapeutic targets and agents are desirable [6].

Within the osteolytic lesions of bone metastases, tumor cells interact with osteoclasts (bone resorbing cells) and osteoblasts (bone forming cells), thereby inhibiting normal bone development and ultimately leading to bone destruction [1-4]. As for osteoclasts, their interaction with tumor cells is reciprocal: tumor cells produce factors (e.g., parathyroid hormone-related peptide; interleukin-6; tumor necrosis factor; and macrophage colony stimulating factor, M-CSF) that directly or indirectly induce the formation of osteoclasts, and activated osteoclasts produce factors (e.g., transforming growth factor, TGF-β; insulin growth factor, IGF; and bone morphogenetic proteins, BMPs) that stimulate tumor growth and bone destruction [1]. Despite a general comprehension of this process, we are still far from a complete mechanistic understanding and lack well defined targets for therapeutic intervention.

Several animal models have been developed to study the mechanisms governing cancer-mediated osteolysis. However, there is no single animal model that ideally replicates the entire metastatic process from primary breast tumor to bone metastasis. Nevertheless, several models that represent various aspects of bone metastasis have been used successfully to study specific features of the disease. For example, Arguello, et al. developed a model in which melanoma cells injected into the left ventricle of the heart ultimately form bone metastases [7]. This model was later used to study various mechanisms behind breast cancer-specific osteoclast formation and bone metastasis [8-10]. Our group has also developed a rat model to study bone metastatic microenvironment in which prostate tumors were directly transplanted onto the calvariae of syngeneic animals. These tumors exhibited pathological osteoblastic and osteoclastic changes [11]. More recently, we used this approach with mouse breast cancer cell lines and found that the tumor cells induce osteolytic changes in the bone microenvironment [12-15]. With this model, we found that cathepsin G cleaves the receptor activator of nuclear factor-B ligand (Rankl) leading to enhanced activation of osteoclasts in the breast cancer bone microenvironment [15]. Furthermore, we also demonstrated the importance of TGF-β signaling and osteoclast activation in the breast cancer bone microenvironment [12,14]. While this series of observations has furthered our understanding of the mechanisms underlying osteolysis, their relevance to human breast cancer remained unknown.

To address this question, we reanalyzed gene expression profiles generated from our previous studies using the syngeneic mouse model of breast cancer specific osteolysis that was developed by implanting 3 different cell lines - 4T1 (highly metastatic), Cl66 (moderately metastatic) and Cl66-M2 (low metastatic) - onto the calvariae bone of BALB/C mice [12-15]. The gene expression profiles were generated from microdissected tumors in which the tumor-bone (TB) interface and the tumor alone (TA) area were isolated independently. Then we identified a TB signature involved in bone destruction by comparing the gene expression profiles of the TA area and TB interface from the dissected tumors. Lastly, using our TB signature, open-access gene expression data, and pathway analytics, we demonstrated that our model mimics human disease and predicted key pathways and a potential therapeutic agent for breast cancer osteolysis.

## Methods

### Mouse osteolytic model and microarray

Mouse breast cancer cell lines - 4T1, Cl66 (66Cl4) and Cl66M2 - with different metastatic potential [16-18] were maintained in culture and were implanted under the dorsal skin flap onto the calvaria of female BALB/c mice, as described [12]. Mice were euthanized and necropsied to examine osteolytic lesions at 4 weeks post implantation. The tissues for histological examination were prepared as described [12]. All studies were carried out in accordance with the Institutional Animal Use and Care Committee (IAUCC) of the University of the Nebraska Medical Center. Calcified frozen tissues were serially sectioned into 10 μm slices and then microdissected to separate the TB interface from the TA area. RNA isolation and gene expression profiling of the TB interface and TA area were performed using Affymetrix GeneChip^® ^Mouse Genome 430A 2.0 Array, as described [14].

### Analysis of gene arrays and public microarray datasets

The CEL files for all the samples from Affymetrix GeneChip^® ^were processed and MAS 5.0-normalized using the SimpleAffy [19] program and robust multiarray (RMA)-normalized using BRB Array tools [20]. The log_2 _MAS 5.0-normalized data was used for subsequent analyses. Fold-change at the TB interface with respect to the TA area for tissues, standard deviation (SD) across TA samples, and median-centered analysis within the TA area were calculated for each of the cell lines (4T1, Cl66 and Cl66-M2) to identify genes up- and down-regulated in the respective samples. The genes were ranked from highest to lowest expression based on the values from fold-change or median-centered analysis.

The following publicly available Affymetrix microarray data were obtained from Gene Expression Omnibus (GEO) [21]: GSE13563 for normal bone from mouse calvaria (n = 2), mandible (n = 2) and ulna (n = 2); GSE14017 (n = 29) and GSE14018 (n = 36) for metastases from breast cancer [22]; GSE11259 (n = 9) for 4T1 primary tumor data [23]; and GSE17563 (n = 3) for osteoclast precursors treated with human RANKL at different time points [24]. All the GEO data were processed and normalized as described above. Affymetrix microarray data for breast tumors (n = 118) [25] and cancer cell lines (n = 54) [26] were also compared with the TA area gene expression profile.

The NearestTemplatePrediction algorithm (NTP) [27,28] was used to predict the class of a given sample with statistical significance (false discovery rate, FDR < 0.2) using a predefined set of markers that are specific to multiple classes (TB interface vs. TA area). Microarray data from different studies and platforms were sample- and gene-normalized and then pooled using the Distance Weighted Discrimination (DWD) algorithm, as described [29,30]. The significance of expression between the mouse model and human bone metastases was estimated using SubMap [31]. Hierarchical clustering of genes and samples were performed using the Cluster software [32]. Visualization was performed with TreeView [32] and Hierarchical Clustering Viewer from GenePattern [33].

### Gene ontology (GO) and pathway analysis

The association of gene signature with known pathways was determined using gene ontology (GO) [34], pathways from Kyoto Encyclopedia of Genes and Genomes (KEGG) [35], and Broad Institute based Molecular Signature Databases (MSigDB) [36]. The enrichment analysis was performed using the TB signature and the GlobalTest package (Version 4.2.0) [37,38].

### Connectivity Map analysis

Gene symbols were mapped to HG-U133A array probes. They were then used to query the Connectivity Map database [39].

## Results

### The TA area resembles the primary tumor

Previously, we transplanted three breast cancer cell lines - 4T1 (highly metastatic), Cl66 (moderately metastatic) and Cl66-M2 (low metastatic) - onto the calvarial bone of BALB/C mice [12,14]. Irrespective of the cell lines used, histochemical analysis of these tumors demonstrated that they exhibited tumor-induced osteolysis and osteoclast activation similar to that observed in breast cancer bone metastasis [12,14]. Metastatic lesions from the osteolytic tumors were microdissected into two cohorts - TB interface and TA area - and gene expression profile analyses were performed [12,14]. Herein, we reanalyzed these gene expression data sets in search of a breast cancer osteolysis-specific gene signature.

**Figure 1:**
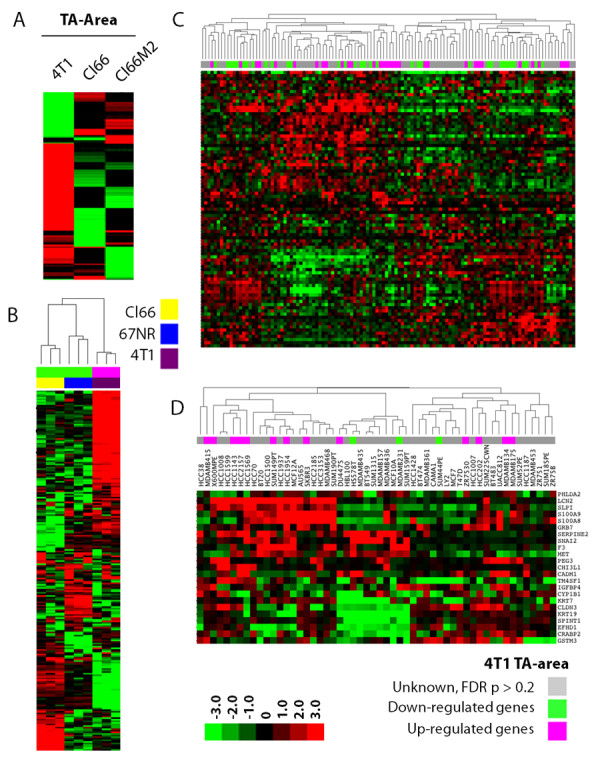
**The TA area resembles matched primary tumors, human breast tumors and cancer cell lines**. **A**. Hierarchical clustering of median centered gene expression from the TA area of the three cell lines (4T1, Cl66 and Cl66M2). **B**. The gene expression profile of primary syngeneic tumors derived using the 4T1, CI66 and 67NR mouse cell lines [23] resembled the TA area in our osteolytic breast cancer model as predicted by NTP algorithm (FDR < 0.2). Primary tumor samples and prediction significance (FDR < 0.2) are color coded and displayed at the top of the hierarchical cluster. **C**. The gene expression profile of primary human breast tumors [25] and **D**. human breast cancer cell lines resembled the TA area of either the 4T1 (up-regulated genes from 4T1 TA area, top bar in pink) or Cl66 (down-regulated genes from 4T1 TA area, top bar in green) of the osteolytic breast cancer model as predicted by NTP algorithm. Prediction significance (FDR < 0.2) for each sample is shown in the colored bar on the top of the hierarchical cluster. Samples predicted with FDR p > 0.2 do not resemble either the TA area or the TB interface.

Our reanalysis illustrates that there is little similarity in gene expression in the TA area samples among the three cell lines (Figure 1A). This is altogether not too surprising given that these cell lines were originally derived from different mouse tumors [16-18]. Consistently, the sublines Cl66 and Cl66-M2 [16-18], share the most similarity in gene expression (Figure 1A).

The TA area was grown in a non-canonical tumor microenvironment (i.e., bone instead of breast) and as such can be considered a metastatic tumor. Nevertheless, we still expect that the gene expression profile from the TA area (grown near bone) will resemble previously reported profiles for the cell lines (grown in the breast) used in this study, especially given the fact that the primary tumor and its metastatic tumor have been reported to have similar gene expression profiles [40]. To confirm that the TA area expression signature of each cell line resembles that of primary tumors, we used a public gene expression profile of tumors grown in the breast from the 4T1 and Cl66 (66Cl4) cell lines [23]. Reassuringly, the up-regulated genes from the TA area of 4T1 cells significantly (FDR p < 0.2) predicted primary tumors from 4T1 cells and the down-regulated genes predicted tumors from Cl66 using the NTP algorithm (Figure 1B). Since the gene signature from the TA area of 4T1 cells are reported relative to Cl66 and Cl66-M2, most of the down-regulated genes represent those up-regulated in Cl66 and Cl66-M2. These results demonstrate that the gene expression profile from our microdissected TA area samples represents that of primary tumors.

In an effort to translate our findings from our mouse breast tumor model to human disease, we compared the gene expression profile from the TA area of our mouse model to that of primary human breast tumors and cancer cell lines using the NTP algorithm. Specifically, we compared microarray data from 118 primary breast tumor samples [25] to the gene expression profile from the 4T1 and Cl66 TA areas. Interestingly, 37 breast tumor samples (top bar in pink) were significantly associated with 4T1 TA area and 34 breast tumor samples were significantly associated with Cl66 TA area (top bar in green) with an FDR p < 0.2 (Figure 1C). Our analysis also predicted that 16 (top bar in pink) and 3 (top bar in green) out of 54 human breast cancer cell lines [26] resemble 4T1 and Cl66 tumors, respectively (Figure 1D). Again, the down-regulated TA area genes represent the TA area of Cl66 and Cl66-M2. This analysis predicts that it is possible to use these 19 human breast cancer cell lines in our mouse model and that similar results could be obtained.

### TB interface-specific gene expression signature

**Figure 2:**
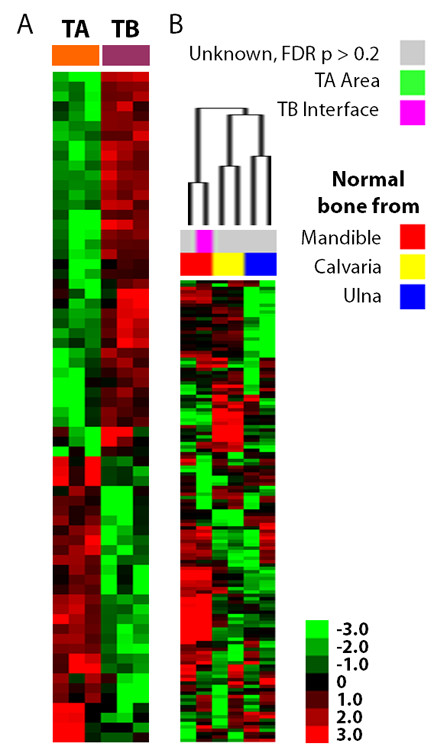
**The TB interface gene signature does not resemble normal bone**. **A**. Top genes differentially expressed at the TB interface common to all the three cell lines. Data are expressed in fold-change relative to TA area: (up-regulated genes (≥2-fold) are shown in red, and down-regulated genes (≤-2-fold) are shown in green. **B**. The NTP algorithm was used to predict whether normal calvarial bone, ulnar bone and mandiblular bone resemble the TB interface of our mouse osteoclastic model. Samples predicted with FDR p > 0.2 do not resemble either the TA area or the TB interface. Samples and prediction significance are shown on the top of the hierarchical cluster.

In order to identify genes that are important for the interaction of breast cancer cells with the tumor microenvironment, we reanalyzed the gene expression at the TB interface and compared that profile to the gene expression profile at the TA area for each of the cell lines. Despite the expected heterogeneity in gene expression from cell line to cell line, we were able to identify 934 genes (TB signature) that were consistently different between the TB interface and the TA area. Among these, 359 were up-regulated and 575 were down-regulated with at least a 2-fold change at the TB interface across all the three cell lines. Figure 2A illustrates the top 50 known up- and down-regulated genes. The top differentially expressed genes are detailed in Tables 1 and 2.

**Table 1:** Genes up-regulated in the TB interface and their fold-change relative to the TA area.

Gene	Affymetrix probe ID	Description	Fold change	p- value	FDR p- value
Ibsp	1417484_at or 1417485_at	Integrin binding sialoprotein	7.2	0.05	0.2

Tnfsf11 (Rankl)	1419083_at or 1451944_a_at	Tumor necrosis factor (ligand) superfamily, member 11	5.3	0.006	0.1

Aftph	1426861_at	Afitiphilin	4.8	0.05	0.2

Mmp13	1417256_at	Matrix metalloproteinase 13	4.7	0.05	0.2

Anks1b	1447464_at	Ankyrin repeat and sterile alpha motif domain containing 1B	4.4	0.02	0.1

Zic1	1423477_at or 1439627_at	Zinc finger protein of the cerebellum 1	3.7	0.09	0.2

Drd1a	1455629_at or 1456051_at	Dopamine receptor 1A	3.6	0.005	0.1

Alpk2	1452478_at	Alpha-kinase 2	3.2	0.005	0.1

Smoc2	1415935_at or 1431362_a_at	SPARC related modular calcium binding 2	3.2	0.01	0.1

**Table 2:** Genes down-regulated in the TB interface and their fold-change relative to the TA area.

Gene	Affymetrix probe ID	Description	Fold change	p-value	FDR p-value
Cnpy1	1437996_s_at	Canopy 1 homolog (Zebrafish)	-5.0	0.09	0.2

Adora3	1429609_at or 1430482_at	Adenosine A3 receptor	-4.7	0.04	0.2

Tmco5	1420341_at	Transmembrane and coiled-coil domains 5	-4.5	0.1	0.2

V1ra2	1427675_at	Vomeronasal 1 receptor, A2	-4.4	0.07	0.2

Maf	1435828_at	Avian musculoaponeurotic fibrosarcoma (v-maf) AS42 oncogene homolog	-3.9	0.02	0.1

Dll3	1449236_at	Delta-like 3	-2.9	0.04	0.2

Krtap16-1	1425655_at	Keratin associated protein 16-1	-2.9	0.01	0.1

Camta1	1433971_at	Calmodulin binding transcription activator 1	-3.5	0.01	0.1

The gene expression profile of the TB interface was identified relative to the TA area, and, as such, should be enriched for transcriptional processes associated with the TB microenvironment. Indeed, three of the top four genes up-regulated at the TB interface (i.e., receptor activator of NF-kB ligand, Rankl; integrin binding sialoprotein, Ibsp; and matrix metalloproteinase 13, Mmp13) are well established as mediators of bone metastasis [7,11-13,15,41,42]. Table 1 highlights the fold-change of these genes at the TB interface as compared to the TA area (from the Affymetrix microarray profiling). Furthermore, we have previously validated the expression and function of several of these genes in our mouse model [12-15]. Collectively, these data strongly suggest that our analysis identified genes uniquely enriched in and important for the metastatic bone microenvironment.

### The TB microenvironment is different than normal bone

Next, we compared the specificity of our TB specific gene set against that from the normal bone microenvironment. To this end, we used a public gene expression profile containing data for normal mouse calvarial bone, normal mouse ulnar bone and normal mouse mandibular bone (GSE13563). Our TB signature was compared against this data set using the NTP algorithm. As shown in Figure 2B, none of the calvarial or ulnar samples are enriched for the TB-signature (FDR p > 0.2), though one of the mandibular bone samples is predicted to be similar to TB microenvironment. This data demonstrates that the TB interface is genetically different from the microenvironment of normal bone.

### The TB interface resembles the metastatic bone microenvironment of human breast cancer

**Figure 3:**
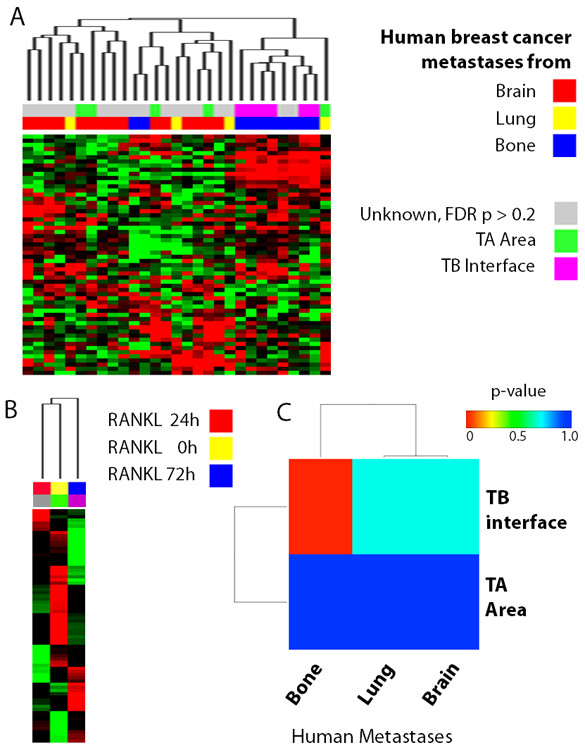
**The TB interface gene signature resembles human bone metastases and osteoclastogenesis**. **A**. The NTP algorithm was used to predict whether human breast cancer metastases (lung, bone and brain) significantly (FDR < 0.2) resemble the TB interface of our mouse osteoclastic model. **B**. SubMap based *de novo *analysis was used to compare the TB interface and TA area gene expression profiles to human breast cancer metastases samples. p-values are color coded in the bar. **C**. The NTP algorithm was used to predict whether the gene expression profile of the TB interface resembles osteoclasts (72 h RANKL treatment; blue) or OCPs (0 and 24 h RANKL treatment; yellow and red, respectively). Samples predicted with FDR p > 0.2 do not resemble either the TA area or the TB interface. Samples and prediction significance are shown in the colored bar on the top of the hierarchical cluster.

A primary concern with any animal model is whether it accurately represents human disease. To address this, we applied NTP using the TB signature and publicly available gene expression profiles of human breast metastases (i.e., brain, lung and bone) [22]. As shown in Figure 3A, 60% of the samples from bone metastases were significantly predicted (FDR p < 0.2) to belong to the TB interface of our model. Importantly, the gene expression profiles of metastases from both brain and lung did not correlate with the TB interface data.

In addition, we also performed the Gene Set Enrichment Analysis (GSEA) [36] based SubMap algorithm [31] to predict if the TB interface gene expression profile resembles bone metastases from humans. Here, SubMap analysis with the TB signature was used to compare different human metastases samples (brain, bone and lung) to the sample sets from our mouse model (TB interface and TA area). Interestingly, *de novo *analysis showed that TB interface samples significantly (FDR < 0.2) resemble bone metastases samples but not lung or brain samples. TA area samples also do not resemble any of the metastases (Figure 3B). Furthermore, the Rankl and Mmp13 genes, which are up-regulated at the TB interface, are also up-regulated in the human bone metastases samples. Collectively, these data demonstrate that the osteolytic bone microenvironment in our mouse model mimics the bone microenvironment in human breast cancer but not that of other metastatic microenvironments (i.e., lung and brain metastases).

### The TB interface resembles osteoclastogenesis in culture

The Rankl-mediated differentiation of osteoclast precursors (OCPs) to mature osteoclasts is a key step in breast cancer-specific bone metastasis [43]. Since Rankl is among the most highly up-regulated genes at the TB interface, we suspected that osteoclastogenesis may be occurring at the TB interface in our mouse model. To address this possibility, we performed NTP analysis using our TB signature and a publicly available gene expression profile from OCPs that have been differentiated into osteoclasts *in vitro *[24]. The osteoclasts used in the aforementioned data set were generated following a two stage differentiation protocol: OCPs were pretreated with macrophage colony stimulating factor (M-CSF) and then treated with human RANKL for 0, 24 or 72 h. Terminal osteoclast differentiation requires at least 72 h of RANKL treatment [24]. NTP analysis of our TB signature predicted that it was similar to OCPs treated with RANKL for 72 h with a FDR of p = 0.2. Interestingly, our TB signature did not correlate with either RANKL-untreated OCPs or those only treated for 24 h (Figure 3C). This analysis suggests that osteoclastogenesis is occurring at the TB interface in our model.

### Pathways associated with the TB interface

**Figure 4:**
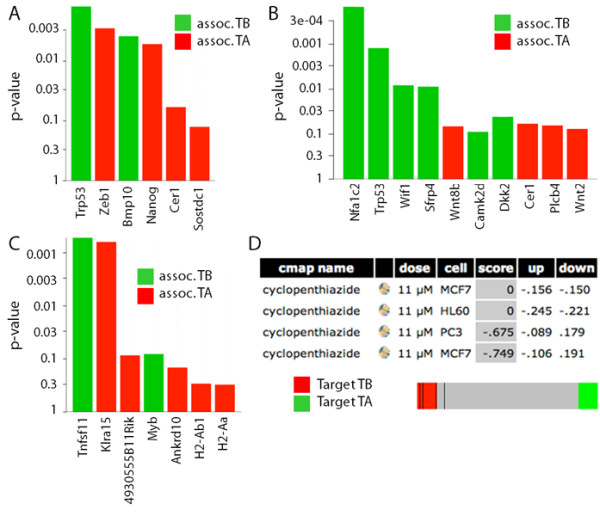
**Genes involved in pathways related to the TB interface and the TA area and prediction of a therapeutic agent that targets the TB interface**. p-values of pathway-specific genes enriched in the TB signature and in the TA area are plotted in green and red, respectively. Differential expression of genes in: **A**. the TGF-β pathway as determined using the GO database; **B**. the Wnt-signaling pathway using the KEGG pathway database; and **C**. the myeloid proliferation and self-renewal pathway [46] using MSigDB. All the enrichment analyses were done using GlobalTest package software. **D**. Connectivity Map analysis predicted cyclopenthiazide as a candidate drug against the TB interface gene signature with 4 instances. All four instances fall in or near the red area (represented by black lines in the bar on the bottom), which suggests that cyclopenthiazide reverses the TB interface gene expression signature.

To assess whether mechanisms that govern bone metastasis in humans are also present in our osteolytic model, we performed Gene Ontology (GO) [34]; pathway Kyoto Encyclopedia of Genes and Genomes, KEGG [44]; and Broad Institute based Molecular Signature Databases, MSigDB [45] canonical pathway enrichment analysis. The enrichment analysis was performed using the TB signature and the GlobalTest package [37,38]. Table 3 shows GO terms significantly (FDR p < 0.05) associated with our osteolytic model. Among the GO terms significantly associated with the TB signature is TGF-β signaling (Figure 4A). Indeed, the TGF-β superfamily ligand Bmp10 is up-regulated at the TB interface in all three cell lines (greater than 2-fold in Cl66 and Cl66M2; data not shown). This would suggest that TGF-β superfamily signaling is mediated in part by the Bmp10 ligand in our model. Consistently, negative regulators (Sostdc1 and Cer1) of the TGF-β pathway are down-regulated at the TB interface and up-regulated in TA area (Figure 4A). These data suggest that Bmp-10 mediated TGF-β superfamily signaling is active at the TB interface but not in the TA area. Future studies specifically over-expressing and knocking-down members of the TGF-β signaling pathway will be required to specifically determine the role of TGF-β signaling at the TB interface.

**Table 3:** GO terms significantly associated with TB signature.

GO ID	Alias	BH	p-value	Statistic
GO:0007219	Notch signaling pathway	0.001	2.65e-05	78.7

GO:0007155	Cell adhesion	0.001	3.39e-06	56.3

GO:0022610	Biological adhesion	0.001	3.39e-06	56.3

GO:0007178	Transmembrane receptor protein serine/threonine kinase signaling pathway	0.001	3.63e-06	77.0

GO:0007249	I-kappaB kinase/NF-kappaB cascade	0.001	4.49e-05	93.6

GO:0005164	Tumor necrosis factor receptor binding	0.001	4.49e-05	93.6

GO:0002761	Regulation of myeloid leukocyte differentiation	0.005	0.00052	96.3

GO:0045637	Regulation of myeloid cell differentiation	0.005	0.00052	96.3

GO:0007179	Transforming growth factor beta receptor signaling pathway	0.008	0.0014	93.5

GO:0017015	Regulation of transforming growth factor beta receptor signaling pathway	0.008	0.0014	93.5

Pathways identified using KEGG analysis that were significantly (FDR p < 0.05) associated with our osteolytic model are shown in Table 4. Interestingly, the Wnt-signaling pathway is significantly associated with the TB signature (Figure 4B), and it appears to be inhibited. Indeed, two Wnt pathway antagonists (Wif1 and Sfrp4) are expressed greater than 2-fold at the TB interface for all the mouse cell lines (data not shown). Among the four most down-regulated genes at the TB interface, relative to the TA area, two are Wnt pathway agonists (Wnt8b and Wnt2). These data suggest that the Wnt signaling pathway is active in the TA area but inhibited in the TB interface. Again, future studies specifically over-expressing and knocking-down members of the Wnt signaling pathway may be performed to further elucidate the role of Wnt signaling at the TB interface and in the TA area.

**Table 4:** KEGG pathways significantly associated with TB-signature.

KEGG ID	Alias	p-value	BH	Statistic
04060	Cytokine-cytokine receptor interaction	7.60e-05	0.003	75.3

01100	Metabolic pathways	8.88e-05	0.003	52.8

05016	Huntington's disease	9.74e-05	0.003	57.9

04310	Wnt signaling pathway	1.07e-04	0.003	72.0

00061	Fatty acid biosynthesis	1.26e-04	0.003	98.2

04912	GnRH signaling pathway	1.26e-04	0.003	57.6

04020	Calcium signaling pathway	3.59e-04	0.007	67.1

05010	Alzheimer's disease	4.15e-04	0.007	66.5

04650	Natural killer cell mediated cytotoxicity	5.35e-04	0.008	60.9

04012	ErbB signaling pathway	6.28e-04	0.009	59.9

We also performed enrichment analysis of the TB signature using MSigDB canonical pathway database and GlobalTest package [37,38]. Among the pathways significantly associated with the TB interface (Table 5) were myeloid proliferation and self-renewal [46]. Consistently, two genes (Rankl and Myb) highly expressed at the TB interface were significantly associated with this pathway (Figure 4C) [45]. This data further corroborates the NTP analysis comparing osteoclasts to our TB signature (Figure 3C) and provides additional evidence for a role of osteoclastogenesis at the TB interface.

**Table 5:** MSigDB pathway signatures significantly associated with TB signature.

Alias	p-value	BH	Statistic
INTRINSICPATHWAY	0.0004	0.0007	96.9

BLOOD_CLOTTING_CASCADE	0.0004	0.0007	96.9

HSIAO_LIVER_SPECIES_GENES	0.0004	0.0007	96.9

TPA_SENS_MIDDLE_DN	0.0004	0.0007	96.9

TPA__SENS_LATE_DN	0.0004	0.0007	96.9

TPA__SENS_EARLY_DN	0.0004	0.0007	96.9

HSA04610_COMPLEMENT_AND_COAGULATION_CASCADES	0.0004	0.0007	96.9

BROWN_MYELOID_PROLIF_AND_SELF_RENEWAL	0.0062	0.010	60.2

BROWN_GRAN_MONO_DIFFERENTIATION	0.0121	0.017	38.9

KAMMINGA_EZH2_TARGETS	0.0521	0.068	65.2

### Prediction and validation of therapeutic targets using the TB signature

To predict a therapeutic agent that specifically targets the TB interface, we queried Connectivity Map database [39] using the TB gene signature. Probeset identifiers from the Affymetrix Mouse Genome 430A 2.0 array were mapped to Affymetrix Human Genome U133A array. This was then used to query the Connectivity Map database. Of the 6,100 potential therapeutic candidates, cyclopenthiazide had the most highly significant negative mean connectivity scores. In other words, cyclopenthiazide was predicted to reverse the gene expression signature of the TB interface (Figure 4D). This analysis suggests that cyclopenthiazide may be a potential agent against human osteoclastic bone metastasis. Future studies aim to address this possibility by therapeutically dosing our mouse model with cyclopenthiazide and monitoring for changes in the TB microenvironment.

## Discussion

### Mouse Model of the Osteolytic Microenvironment in Breast Cancer

Animal models that faithfully recapitulate aspects of human breast cancer-specific bone metastasis provide powerful tools to study the complex molecular mechanism(s) by which breast cancer cells metastasize to and interact with the bone microenvironment [47,48]. Previously, we developed mouse models of bone osteolysis for prostate and breast cancer by implanting syngeneic tumor cells onto the calvaria of animals using a simple surgical technique. These models produced osteolytic lesions at the TB interface of the implant region, thereby allowing us to explore the cellular and molecular interactions between malignant cells and skeletal tissue [11,12,15]. Because the tumor cells are implanted directly into the bone microenvironment (albeit, at an atypical location for breast cancer bone metastasis), it was important to confirm that the interactions observed in our model reflect those observed between metastatic human breast cells and the bone microenvironment. Building on our previous work, we now demonstrate that the TB microenvironment in our model appears very similar to that of human breast cancer bone metastases based on gene expression data. As such, this mouse model can be readily used to study the cellular and molecular mechanisms driving human breast cancer-metastasis and osteolysis. Furthermore, this model also provides a powerful preclinical setting to test cyclopenthiazide and other therapeutic agents that specifically target breast cancer osteolysis.

### Gene Expression Profile Analysis

There has been tremendous growth in both the development of high-throughput microarray technology to measure gene expression in tissue and cells and in high-dimensional methods to analyze such data [49,50]. Together with this, many of the gene expression microarray data sets generated from different labs are now available in open-access databases [21,51], which enables the comparison and integration of data acquired from different batches, laboratories and experimental platforms [52]. Importantly, this has opened up opportunities to perform cross-species comparisons of mouse models and human disease [30].

In the current study, we applied microarray technology to generate a signature specific to the TB interface of our mouse model. The robustness of our TB-signature is supported by the fact that it was derived from a common set of genes regulated at the TB interface across a heterogeneous set of three mouse breast cancer cell lines. Combining gene expression profiling and molecular pathology, we demonstrated that the TB interface of our model truly represents the tumor microenvironment and not the normal bone microenvironment. Subsequent cross-species comparative transcriptomic analysis demonstrated that many human bone metastases samples are associated with the TB interface in a statistically significant manner. Importantly, there was no association between our breast TB interface and human brain or lung metastases. Together, these data demonstrate that our model specifically mimics human breast (and not lung or brain) cancer bone metastases. Furthermore, analysis of a panel of human breast cancer cell lines predicted 16 that have similar gene expression characteristics to those of the 4T1 tumors. This suggests that our osteolytic model may be adapted to study human breast cancer bone metastasis directly using any of these 16 human cell lines.

### Pathways involved in the Breast Cancer Osteolytic Microenvironment

The TGF-β pathway has a well established role in bone metastasis [53], and previously we demonstrated the importance of TGF-β signaling in the TB interface using our model [12]. Here, we demonstrate that the TGF-β receptor I is expressed and that the TGF-β pathway is active in tumor cells and osteoclasts at the TB interface. On the other hand, TGF-β signaling is not active in the TA area [12]. Interestingly, the TGF-β signaling ligand Bmp10 [54] is highly expressed at the TB interface and TGF-β pathway inhibitors (i.e., Sostdc1 [55] and Cer1 [56]) are suppressed at the TB interface. These data suggest that Bmp-10 is responsible for mediating TGF-β pathway activation at the TB interface.

The canonical and noncanonical Wnt signaling pathways are involved in the formation, growth and development of normal bone [57] and bone metastasis [58]. Activation of canonical Wnt signaling through β-catenin both promotes osteoblast differentiation and inhibits osteoclast formation and bone resorption [59]. Our KEGG pathway enrichment analysis showed a significant association of the Wnt signaling pathway at the TB interface. Indeed, we observed that Wnt pathway antagonists - Wif1, which is associated with decreased bone mineral density [60], and Sfrp4, which is associated with the suppression of osteoblast proliferation [61] - were over-expressed at the TB interface. Furthermore, we observed a down-regulation of the Wnt pathway ligands Wnt2 [62] and Wnt8b [63] at the TB interface relative to the TA area. Together these data suggest that our mouse model exhibits (i) Wnt pathway activation in the TA area and (ii) increased bone resorption and suppressed bone formation (at least in part through Wnt pathway activation) at the TB interface.

Osteoclasts are derived from hematopoietic precursor cells of the myeloid lineage upon CSF-1 stimulation followed by RANKL-mediated maturation [43]. In our current study, we used a publicly available microarray dataset from RANKL-differentiated OCPs. Interestingly, we found that the gene expression profile of *in vitro *differentiated osteoclasts (72 h RANKL treatment) was similar to that of the TB interface. In addition, pathway analysis using the MSigDB showed an enrichment of the TB-signature in a myeloid cell line model [46] (Figure 4C). Overall, these results suggest that osteolysis is operative at the TB interface of our mouse model.

### Prediction of a Therapeutic Agent that Targets the TB interface

The identification of new therapeutic agents that inhibit the establishment of tumor cells in the TB microenvironment will benefit patients with breast cancer bone metastases [5]. This will require a thorough understanding of the mechanisms governing breast-to-bone metastasis to determine appropriate biological targets for intervention. In one example, we previously demonstrated that TGF-β signaling activity may provide such a target as pathway attenuation in our mouse model led to a reduction in breast tumor-induced osteolysis [12]. Herein, we used gene expression profiles from our mouse model and Connectivity Map database to find therapeutic agents that target the TB interface, rather than a given pathway.

The advantage of Connectivity Map database is that it can predict potential therapeutic agents based solely on gene signatures [39]. In the current study, our query of Connectivity Map database with the TB signature flagged cyclopenthiazide in the MCF7 cell line (Figure 4D). This analysis suggests that cyclopenthiazide has the potential to inhibit the establishment of breast cancer cells at TB interface.

Thiazides comprise a class of diuretic agents (of which cyclopenthiazide is a member) that are traditionally used to treat hypertension and edema [64]. Although thiazides have not been widely viewed as therapeutic agents for bone metastasis, reports abound noting that treatment of hypertension using thiazides has the beneficial side effect of strengthening bone [65-69]. Furthermore, Devorak *et al*. have demonstrated that the bone strengthening activity of thiazides results from their direct action on OCPs, where thiazide analogs are able to directly induce osteoblast differentiation [70]. These data suggest that cyclopenthiazide may be a useful agent against osteoclastic bone metastasis. Future efforts are aimed at validating this prediction in the osteolytic mouse model. This study serves as an example of how mouse breast cancer-specific osteolytic models and gene expression analysis can be used to identify treatment strategies for human disease.

## Conclusions

In summary, we have demonstrated that the TB microenvironment in our mouse model of osteolytic breast cancer metastasis is highly similar to that of human breast cancer-to-bone metastases. Furthermore, gene expression profile analysis of tumors from this model: (i) identified a TB interface specific gene signature; (ii) revealed signaling pathways that were differentially activated at the TB interface and TA area; (iii) demonstrated a role for osteoclasts in metastatic osteolysis; and (iv) predicted a novel therapeutic agent that specifically targets the TB interface. These data clearly demonstrate that this mouse model can be used to study the cellular and molecular mechanisms driving human breast cancer-to-bone metastasis and osteolysis. Moreover, this model also provides a powerful preclinical setting to test thiazides and other therapeutic agents that specifically target breast cancer osteolysis.

## Competing interests

WJG is currently an employee of Genomic Health.

## Authors' contributions

AS conceived and developed the concept, performed all the analyses and wrote the manuscript. MF performed the animal experiments. CAL contributed to the scientific content. CAL and WJG helped with editing of the manuscript. MF and RKS conceived the idea for the development of the animal model. All authors read and approved the final manuscript.

## Pre-publication history

The pre-publication history for this paper can be accessed here:

http://www.biomedcentral.com/1471-2407/11/304/prepub
